# Three-Dimensional Segmentation Assisted with Clustering Analysis for Surface and Volume Measurements of Equine Incisor in Multidetector Computed Tomography Data Sets

**DOI:** 10.3390/s23218940

**Published:** 2023-11-02

**Authors:** Marta Borowska, Tomasz Jasiński, Sylwia Gierasimiuk, Jolanta Pauk, Bernard Turek, Kamil Górski, Małgorzata Domino

**Affiliations:** 1 Institute of Biomedical Engineering, Faculty of Mechanical Engineering, Białystok University of Technology, 15-351 Bialystok, Poland; m.borowska@pb.edu.pl (M.B.); gierasimiuksylwia@gmail.com (S.G.); j.pauk@pb.edu.pl (J.P.); 2 Department of Large Animal Diseases and Clinic, Institute of Veterinary Medicine, Warsaw University of Life Sciences, 02-787 Warsaw, Poland; tomasz_jasinski@sggw.edu.pl (T.J.); bernard_turek@sggw.edu.pl (B.T.); kamil_gorski@sggw.edu.pl (K.G.)

**Keywords:** diagnostic imaging, image processing, equine dentistry, volumetric feature, horse

## Abstract

Dental diagnostic imaging has progressed towards the use of advanced technologies such as 3D image processing. Since multidetector computed tomography (CT) is widely available in equine clinics, CT-based anatomical 3D models, segmentations, and measurements have become clinically applicable. This study aimed to use a 3D segmentation of CT images and volumetric measurements to investigate differences in the surface area and volume of equine incisors. The 3D Slicer was used to segment single incisors of 50 horses’ heads and to extract volumetric features. Axial vertical symmetry, but not horizontal, of the incisors was evidenced. The surface area and volume differed significantly between temporary and permanent incisors, allowing for easy eruption-related clustering of the CT-based 3D images with an accuracy of >0.75. The volumetric features differed partially between center, intermediate, and corner incisors, allowing for moderate location-related clustering with an accuracy of >0.69. The volumetric features of mandibular incisors’ equine odontoclastic tooth resorption and hypercementosis (EOTRH) degrees were more than those for maxillary incisors; thus, the accuracy of EOTRH degree-related clustering was >0.72 for the mandibula and >0.33 for the maxilla. The CT-based 3D images of equine incisors can be successfully segmented using the routinely achieved multidetector CT data sets and the proposed data-processing approaches.

## 1. Introduction

Medicine, both human and veterinary, is rapidly changing with innovations in medical technology and education. Radiology is one of the areas that should reflect the learning and clinical requirements of doctors in an era of advanced technology such as three-dimensional (3D) image processing and assisted image interpretation [1,2]. In particular, when a surgical removal or replacement of a specific anatomical structure is needed, 3D imaging, such as computed tomography (CT), and anatomical 3D model reconstruction are performed before surgery to identify the best anatomical and spatial conditions [3,4]. The preoperative preparation of the CT-based anatomical 3D model improves the planning of the surgery, shortens the surgery time, and reduces the risk of complications so that both treatment outcomes and communication with the patients [3] or animal’s owners [4] are much better.

In equine dentistry, anatomical 3D modeling was recently used to investigate the physiology of mastication and attrition based on both incisors [5,6] and cheek teeth [7] characteristics. Listmann et al. (2017) and Kau et al. (2020) applied CT-based 3D anatomical models for defining dental landmarks and angulations at different incisor tooth locations [5,6]. Listmann et al. (2017) examined eighteen equine cadaveric heads on a 16-slice fan beam CT scanner to determine the incisor table angles and identify morphological landmarks useful for a further clinical study [6], whereas Kau et al. (2020) revised scans of 48 horses, made on a 16-slice fan beam CT scanner, to investigate age-related changes and incisor tooth location-related differences of interincisal angulation [6]. However, both researchers used detailed 3D models featuring bones of the skull and the dentition concerning the specific 2D landmarks rather than the specific 3D anatomical structures. In anatomical 3D model preparation, the specific 3D anatomical structure may be isolated, visualized, and then measured on the CT data sets by the 3D image segmentation, which involves digitally marking the anatomical regions of interest (ROIs) [1]. In equine dentistry, Herren et al. (2022) applied segmentation to cone beam CT scans of five equine cadaver heads to assess the feasibility of measuring the volume of cheek tooth [7]. To date, no further digital CT-based 3D segmentation studies on equine teeth are available.

One may observe that the head CT is more available in equine clinics [8] since it has become routinely used in diagnostic protocols of i.a. paranasal sinuses [9], nasal cavity [10], and dental diseases [11,12,13]. In horses with suspicious clinical symptoms and normal radiographic signs especially, CT can provide the necessary findings to make a diagnosis. When it is unclear on radiogram whether any dental involvement or more teeth are affected, CT is beneficial [14,15]. Moreover, it can have great value in horses with incomplete remission of clinical symptoms despite medical or surgical treatment [16]. Among dental diseases, calculus, caries, fractures, and loose teeth can affect both the incisor [17] and cheek teeth [18], whereas equine odontoclastic tooth resorption and hypercementosis (EOTRH) affects only the incisor and canine teeth [19]. Calculus of the incisors and caries of the cheek teeth are most commonly reported [20]. Moreover, fractures commonly occur secondary to infundibular caries [21], just as with the apical dental infection that appears usually secondary to pulpar infection, fractures, and abnormally positioned teeth [22]. Abnormal teeth position, as well as other malocclusions, can impair dental and periodontal function [23], and occur more frequently than dental diseases do [20]. Fortunately, in many cases, malocclusion teeth can be individually corrected using standard procedures to maximize their functional occlusion [23].

As equine hypsodont teeth are subject to continuous growth [24], less abrasive forage-favored incisors, and cheek teeth overlength, this results in a lack of occlusal wear [25]. The constant growth of horses’ teeth is also a cause of age-related positional and morphometric alterations of incisors [5] and permanent periodontal remodeling [24]. One also may note that horses have two sets of teeth, one temporary and one permanent [26] and only permanent teeth are hypsodont [27]. From the morphometric point of view, permanent teeth are larger and longer than temporary ones [26]. Thus, the completion of tooth replacement at the age of 3, 4, and 5 years for center, intermediate, and corner incisors, respectively [26], should be considered in the case of dental morphometric and volumetric measurements. Given that recent equipment and room modifications allow for the use of regular multidetector fan beam CT in standing sedated horses [16,28], CT-based anatomical 3D models, segmentations, and measurements have also become clinically applicable in equine dentistry.

In this paper, we hypothesize that the CT-based 3D images of equine incisors can be successfully segmented to obtain morphometric and volumetric features, and the returned data sets enable easy (temporal/permanent), moderate (tooth location and age), and difficult (degrees of dental disease) clustering that may find clinical application in the future. Therefore, the study aimed to use a 3D segmentation of CT images and volumetric measurements to investigate eruption-related, location-related, age-related, and EOTRH degree-related differences in the surface area and volume of consecutive incisors.

## 2. Materials and Methods

### 2.1. Animals and Study Design

This observational, cross-section study was designed based on 50 equine heads obtained in a commercial slaughterhouse. Immediately after slaughter, the heads were transported to the Equine Clinic of the Warsaw University of Life Sciences, where multidetector CT imaging was performed. The heads were collected from warmblood horses and provided with metadata containing the sex and age of the horse so that the entire study population consisted of 29 mares and 21 geldings aged 1 to 30 years (mean ± SD: 9.94 ± 7.91 years).

**Figure 1:**
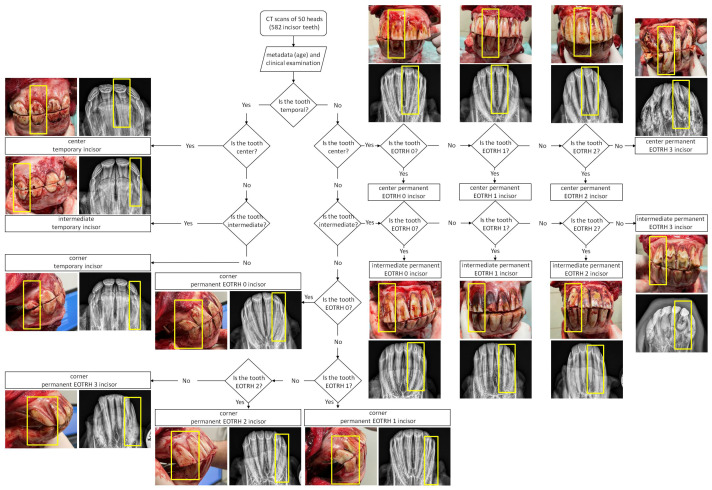
The flowchart for assigning each equine incisor to one of the studied groups. Assignation is based on the results of oral cavity inspection (visible light image) and radiographic imaging (radiogram). An example of an incisor tooth belonging to each group is marked with a yellow frame.

In the Equine Clinic, heads were subjected to detailed dental examinations conducted following the standard protocol [20]. Each examination of the oral cavity was supported by intraoral radiographic imaging of maxillary and mandibular incisors. The radiographic imaging was performed following the standard protocol and under previously described X-ray tube settings [29]. Both examinations provided data for each subsequent tooth on (1) the presence of temporal or permanent teeth, (2) tooth location, (3) signs of EOTRH, as well as (4) signs of calculus, caries, fractures, and loose teeth (Figure 1).

The signs of eruption and the tooth location were recognized following Loch and Bradley (1998) [26], so that the incisors were named center, intermediate, and corner counting from the midline of the maxilla and mandible. The center incisors corresponded to teeth 101, 201, 301, and 401; the intermediate incisors corresponded to teeth 102, 202, 302, and 402; and the corner incisors corresponded to teeth 103, 203, 303, and 403 according to the modified Triadan system [30]. The degree of EOTRH was recognized as normal (0), mild (1), moderate (2), or severe (3) following Hüls et al.’s (2012) classification [31] with Rehrl et al.’s (2018) modification [32] as shown in our previous researches [29,33,34]. The presence of signs of dental disease other than EOTRH was an exclusion criterion. These signs were recognized following guidelines described in Górski et al. (2022) [20]. Due to tooth fracture, two sets of maxillary incisors and one set of mandibular incisors were excluded, so 582 incisors, 288 maxillary incisors and 294 mandibular incisors, were further evaluated (Table 1).

### 2.2. CT Image Acquisition

The CT imaging was performed in a 64-slice CT (Revolution CT, GE Healthcare, Chicago, IL, USA). Scanning was performed using the following imaging parameters: helical scan type GSI 20 mm; current 275 mA; voltage GSI-QC (Dual Energy, variable in the range of 70–140 kV) X-ray tube current; lamp rotation 0.08/s/HE+; table travel 39.4 mm/revolution and table rotation stroke 0.984:1; layer thickness 2.5 mm; spatial resolution 0.23 mm/50 cm SFOV.

The scanning range started through the labial surface of the incisors and ended behind the caudal edge of the occipital bone. The length of the scanned area and the number of scanned layers were adjusted to the size of the scanned head. The obtained images were reconstructed on the AW station using VolumeShare 7 software (GE Healthcare, Chicago, IL, USA) using the following settings: detailed reconstruction (detail); mono voltage 70 keV; ASIR 40%; layer thickness 0.625 mm. Voxel dimensions = pixel size × layer thickness = DFOV/matrix size × layer thickness = 200 mm/512 × 0.625 mm = 0.39 × 0.39 × 0.625. The reconstructed images were saved in DICOM standard.

### 2.3. CT Images Segmentation

The main objective of the segmentation was to extract the anatomical structures of the teeth from the CT scans to create digital 3D models, which were characterized by the total tooth surface area and tooth volume. Segmentation was performed using the open-source software 3D Slicer v4.10 [35]. The 3D Slicer is used in clinical and biomedical applications to perform visualization, segmentation, image processing and analysis, 3D printing planning, and navigation of image-guided procedures. The 3D Slicer is compatible with the DICOM standard, which enables work on medical images saved in this extension.

**Figure 2:**
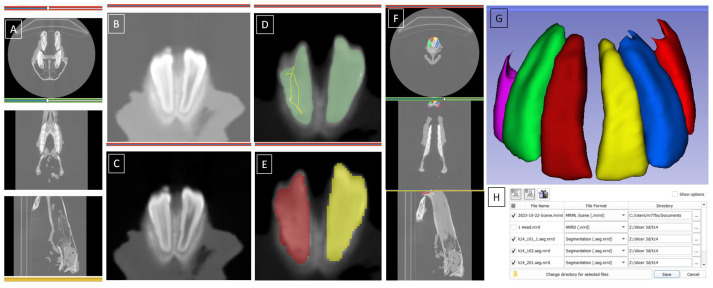
The 3D model of equine incisors. The CT file is processed according to the following steps: loading data in three planes (**A**), interacting with the bone (**B**), adjusting contrast (**C**), thresholding (**D**), creating masks (**E**), smoothing masks in three planes (**F**), outlining segmented incisors in colors in the 3D rotational view (**G**), and exporting segmentation as a digital 3D model in stereolithography file format (**H**).

The DICOM files were inspected, prepared, and processed according to the following steps; (1) loading the DICOM file into 3D Slicer, (2) interacting with the three planes using simple tools (e.g., panning, zooming, and windowing), (3) adjusting contrast, (4) thresholding, (5) creating a mask, (6) smoothing, and (7) exporting segmentation as a digital 3D model in stereolithography file format. The contrast adjusting step was performed using the "adjust level/window tool”. The area with the upper teeth was located and the optimum contrast level was selected automatically. The thresholding step was performed using the “local threshold” tool. The appropriate brightness threshold was automatically selected based on the respective histogram. The mask-creating step was performed using the built-in “paint” tool. A mask was applied both on the image and in the 3D view. The smoothing step was performed using the “smoothing” tool, as after mask application the 3D view showed some defects (errors related to the creation of holes or outliers). Closing (to fill cavities), opening (to remove outliers), and median filtering (to smooth) operations were used to remove these defects. The exporting step was performed to export the segmentation as a label map for the feature extractor as shown on the 3D model in Figure 2.

The Segment statistics module of 3D Slicer software was used to calculate volumetric measures related to the structure of segmentation. Thus the surface area and volume of each incisor were calculated. The values were computed from the binary label map representation of incisors.

### 2.4. Data Analysis

Univariate marginal distributions of surface area and volume were tested independently for each group and each incisor, separately. A Shapiro–Wilk normality test was used.

First, the axial symmetry of the incisors was tested between the two data series. Data sets between the maxilla and the mandible were compared, for each type of incisor separately. The data sets were then compared between the right and left sides, for each incisor type separately. Next, the eruption-related differences were tested between the two data series. Data sets between the temporary and permanent groups were compared, for each type of incisor separately. Then, the location-related differences were tested between the three data series. Finally, the EOTRH degree-related differences were tested between the four data series. The Unpaired *t*-test with Welch’s correction test or the Mann–Whitney test were used to compare two data series. When both data series were normally distributed, the Unpaired *t*-test with Welch’s correction test was used. When at least one data series was not normally distributed, the Mann–Whitney test was used. The Kruskal–Wallis test followed by Dunn’s multiple comparisons test was used to compare more than two data series. The significance level was established as *p* < 0.05. At least one data series for each feature was not normally distributed; therefore, median ± quartiles were used to present numerical data on plots.

The similarity between the right and left sides and age-related changes were tested using linear regression and correlation. The regression equations and coefficients of determination ($r^{2}$) were calculated and displayed on plots. When both data series were normally distributed, Pearson’s correlation (*r*) was calculated. When at least one data series was not normally distributed, Spearman’s correlation (ρ) was calculated. The coefficients were considered significant for *p* < 0.05. The above statistical analyses were performed using GraphPad Prism6 software (GraphPad Software Inc., San Diego, CA, USA).

Finally, data sets representing two volumetric features were clustered using the K-means clustering algorithm. Clustering was processed into a specific number of K classes, where K = 2 for easy (temporal/permanent) clustering, K = 3 for moderate (tooth location) clustering, and K = 4 for difficult (EOTRH degrees) clustering. K centroids, one for each cluster, were defined using the k-means++ method and a measure of the Euclidean distance in order to group nearby points in the feature space. K-means clustering was performed by defining the number K of clusters, randomly defining K centroids, calculating the Euclidean distance between each data point in the space and its closest centroid, recalculating the new centroid of each cluster, and repeating the last two steps until the centroid position does not change. The visualization of the multidimensional feature space divided into classes in the form of two-dimensional plots was made using MDS multidimensional scaling. K-means clustering and class visualization were processed using the Scikit-learn package in Python (https://scikit-learn.org/stable/, accessed on 1 September 2023). The clustering results were evaluated using the classification report from the Scikit-learn.metrics in Python (https://scikit-learn.org/stable, accessed on 1 September 2023). The following classification metrics were calculated: recall, precision, accuracy, and the F1-score based on four class prediction rates True Positive (TP), True Negative (TN), False Positive (FP), and False Negative (FN). Recall was calculated for each class as a measure of quantity reflecting the positive results that were correctly classified as Recall = TP/(TP+FN). Precision was calculated for each class as a measure of quality reflecting the fraction of correctly classified positive results among all positive results as Precision = TP/(TP + FP). The F1-score was calculated for each class as the harmonic mean of precision and recall as F1-score = TP/TP + 1/2(FP + FN), whereas accuracy was calculated for all classes as the fraction of correctly classified results using Accuracy = (TP+TN)/(TP + TN + FP + FN).

## 3. Results

### 3.1. Axial Symmetry of the Incisors

In order to exclude or confirm the possibility of further pooling of incisor volumetric features, a comparison of horizontal axial symmetry (between maxillary and mandibular incisors) and vertical axial symmetry (between successive incisors in the same location) was carried out. Global comparison of volumetric features from corresponding tooth locations of the maxilla and mandible revealed significant differences, whereas corresponding tooth locations of the left and right sides revealed no significant difference.

**Figure 3:**
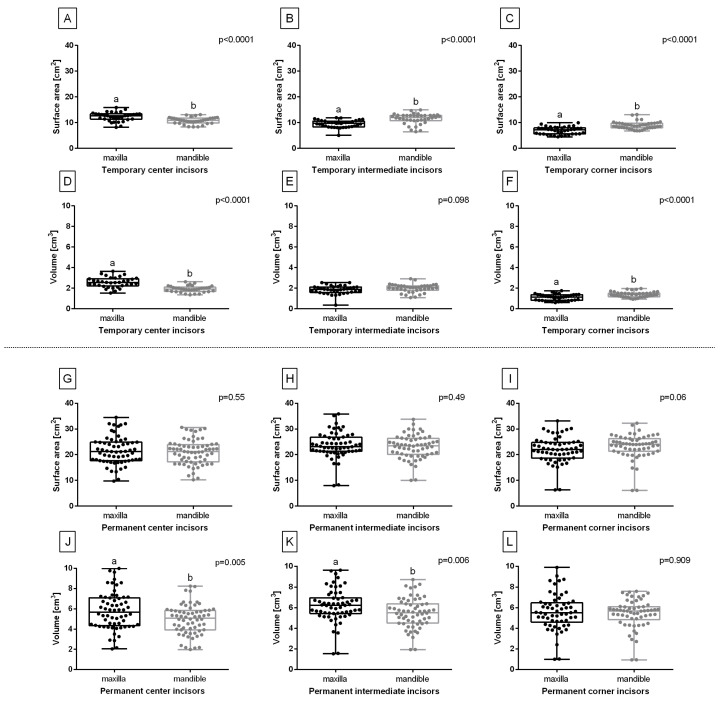
The axial horizontal symmetry compared for the center (**A**,**D**,**G**,**J**), intermediate (**B**,**E**,**H**,**K**), and corner (**C**,**F**,**I**,**L**) incisors. The surface area (**A**–**C**,**G**–**I**) and volume (**D**–**F**,**J**–**L**) of temporary (**A**–**F**) and permanent (**G**–**L**) incisors in the maxilla and mandible. Data in box plots are represented by the lower quartile, median, and upper quartile, whereas whiskers represent minimum and maximum values. Values for subsequent individuals are marked with dots. Lowercase letters indicate differences between groups for *p* < 0.05.

The surface area of all temporary incisors differed between the maxilla and mandible (Figure 3A–C), and the volume of center (Figure 3D) and corner (Figure 3F) incisors also differed between the maxilla and mandible. Although the surface area of all permanent incisors did not vary between the maxilla and mandible (Figure 3G–I), the area of center (Figure 3J) and intermediate incisors (Figure 3K) did. Therefore, the maxillary and mandibular incisors were further considered separately.

**Figure 4:**
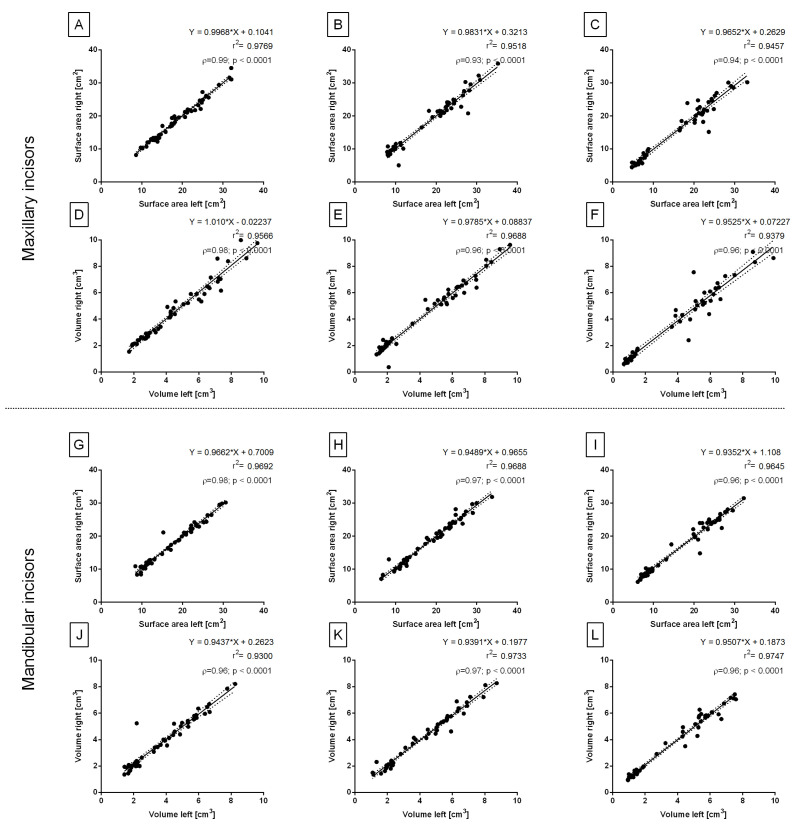
The axial vertical symmetry shown for the center (**A**,**D**,**G**,**J**), intermediate (**B**,**E**,**H**,**K**), and corner (**C**,**F**,**I**,**L**) incisors. Linear regression, Pearson’s correlation (r), and Spearman’s correlation (ρ) for the surface area (**A**–**C**,**G**–**I**) and volume (**D**–**F**,**J**–**L**) of the maxillary incisors (**A**–**F**) and mandibular incisors (**G**–**L**). The regression equations and coefficients of determination ($r^{2}$) are displayed on the plots. Values for subsequent individuals are marked with dots. Dashed lines indicate upper and lower 95% confidence intervals for the regression line. Correlation coefficients are considered significant for *p* < 0.05.

The surface area and volume of all temporary and permanent incisors did not differ between the right and left sides (data not shown). Thus, in the second step of the axial horizontal symmetry determination, the measures of similarity were calculated. For all studied incisors, $r^{2}$ for the right and left data pairs was higher than 0.93 and ρ was higher than 0.93 (Figure 4A–L), which indicates a very strong positive correlation between the sides. Therefore, the incisors of the left and right sides were pooled and further considered jointly.

### 3.2. The Eruption-Related Differences in Incisors’ Volumetric Features

**Figure 5:**
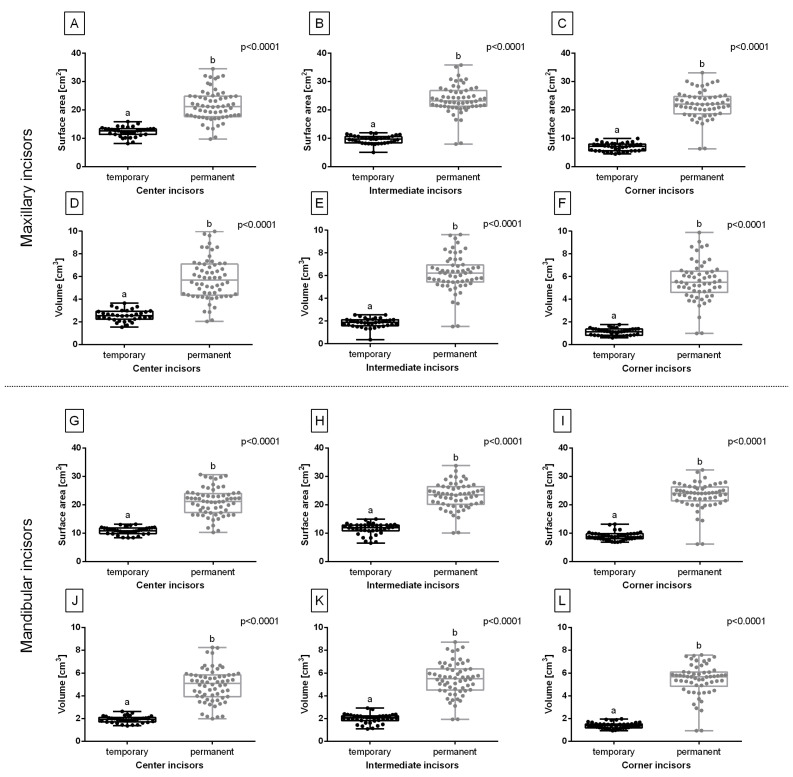
The eruption-related features compared for the center (**A**,**D**,**G**,**J**), intermediate (**B**,**E**,**H**,**K**), and corner (**C**,**F**,**I**,**L**) incisors. The surface area (**A**–**C**,**G**–**I**) and volume (**D**–**E**,**J**–**I**) of maxillary incisors (**A**–**F**) and mandibular incisors (**G**–**L**). Data in box plots are represented by the lower quartile, median, and upper quartile, whereas whiskers represent minimum and maximum values. Values for subsequent individuals are marked with dots. Lowercase letters indicate differences between groups for *p* < 0.05.

The surface area of all maxillary and mandibular incisors was lower for the temporary incisors than for permanent ones (Figure 5A–C,G–I). Moreover, the volume of all maxillary and mandibular incisors was lower for the temporary than the permanent incisors (Figure 5D–F,J–L). For all these reported differences, *p* was <0.0001. Therefore, the eruption-related differences were suspected to be easy for the clustering.

**Figure 6:**
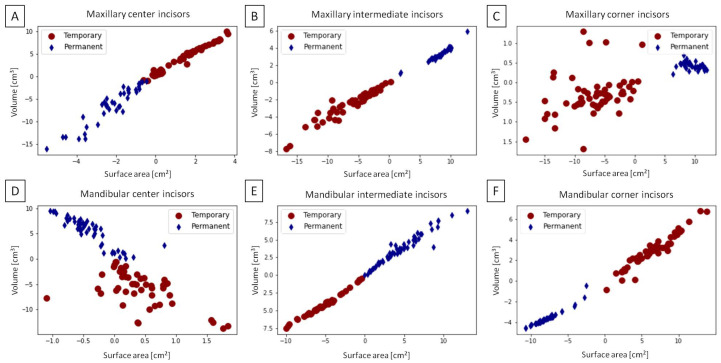
Two eruption-related classes (*Temporary* and *Permanent*) extracted from the volumetric feature space for the center (**A**,**D**), intermediate (**B**,**E**), and corner (**C**,**F**) incisors in the maxilla (**A**–**C**) and mandible (**D**–**F**). Individuals are marked with dots and rhombuses.

The total dataset was clustered as shown in Figure 6 for maxillary center incisors (Figure 6A), maxillary intermediate incisors (Figure 6B), maxillary corner incisors (Figure 6C), mandibular center incisors (Figure 6D), mandibular intermediate incisors (Figure 6E), and mandibular corner incisors (Figure 6D). The accuracy of incisors’ classification was higher for mandibular than maxillary incisors, with the highest accuracy for mandibular intermediate incisors. As all accuracy values were >0.75 (Table 2), the easy eruption-related clustering of the CT-based 3D images of equine incisors was confirmed.

### 3.3. The Location-Related and Age-Related Differences in Incisors’ Volumetric Features

**Figure 7:**
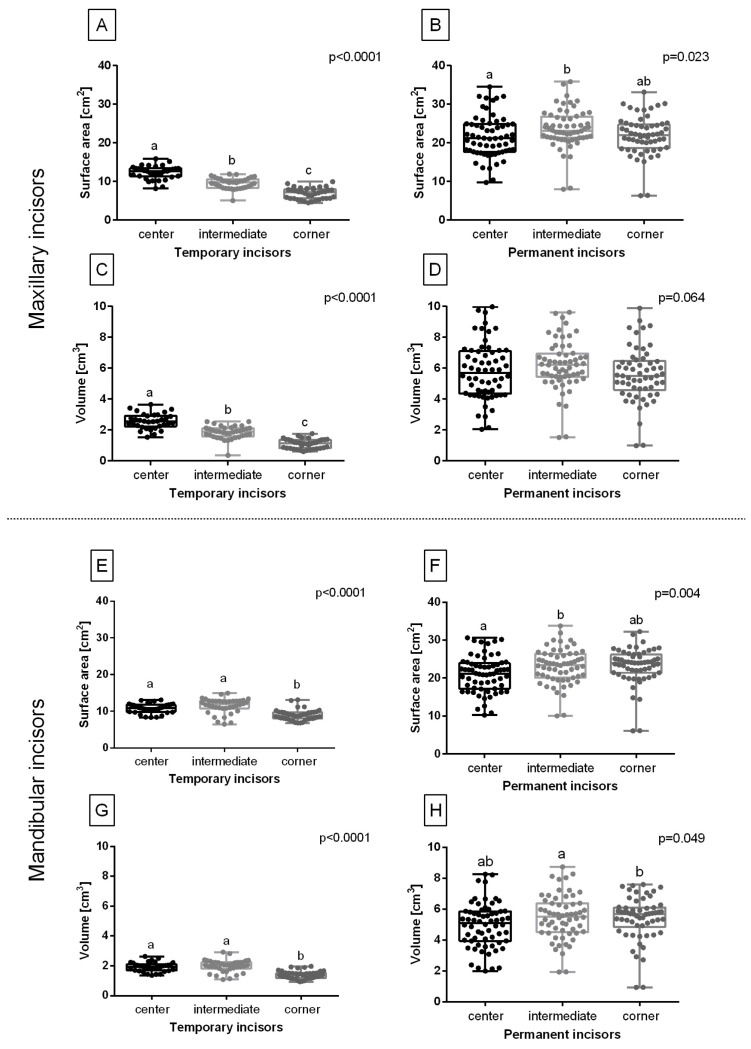
The location-related features compared for the temporary (**A**,**C**,**E**,**G**) and permanent (**B**,**D**,**E**,**L**) incisors. The surface area (**A**,**B**,**E**,**F**) and volume (**C**,**D**,**G**,**H**) of maxillary incisors (**A**–**D**) and mandibular incisors (**E**–**H**). Data in box plots are represented by the lower quartile, median, and upper quartile, whereas whiskers represent minimum and maximum values. Values for subsequent individuals are marked with dots. Lowercase letters indicate differences between groups for *p* < 0.05.

The surface area and volume of temporary maxillary incisors were high for the center incisors, lower for the intermediate incisors, and the lowest for the corner incisors (Figure 7A,C), whereas the surface area and volume of temporary mandibular incisors were higher for the center and intermediate incisors than for the corner ones (Figure 7E,G). On the other hand, the surface area of permanent maxillary and mandibular incisors was lower for the center incisors than for the intermediate ones, with no differences between the center and corner incisors as well as the intermediate and corner incisors (Figure 7B,F). Although the volume of permanent maxillary incisors does not differ between subsequent locations (Figure 7D), the volume of permanent mandibular incisors was higher for the intermediate incisors than the corner ones (Figure 7H). Therefore, the location-related differences, especially for temporary maxillary incisors, were suspected to be moderate for the clustering.

**Figure 8:**
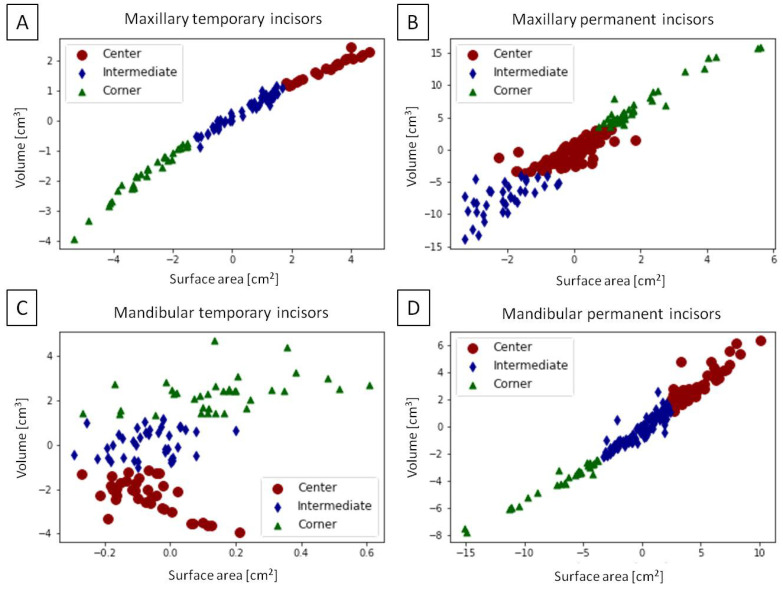
Three location-related classes (*Center*, *Intermediate*, and *Corner*) extracted from the volumetric feature space for the temporary (**A**,**C**) and permanent (**B**,**D**) incisors in the maxilla (**A**,**B**) and mandible (**C**,**D**). Individuals are marked with dots, rhombuses, and triangles.

The total data set was clustered as shown in Figure 8 for maxillary temporary incisors (Figure 8A), maxillary permanent incisors (Figure 8B), mandibular temporary incisors (Figure 8C), and mandibular permanent incisors (Figure 8D). The accuracy of incisors’ classification was higher for temporary than permanent incisors, with the highest accuracy for mandibular temporary incisors. As all accuracy values were >0.69 (Table 3), the moderate location-related clustering of the CT-based 3D images of equine incisors was confirmed.

**Figure 9:**
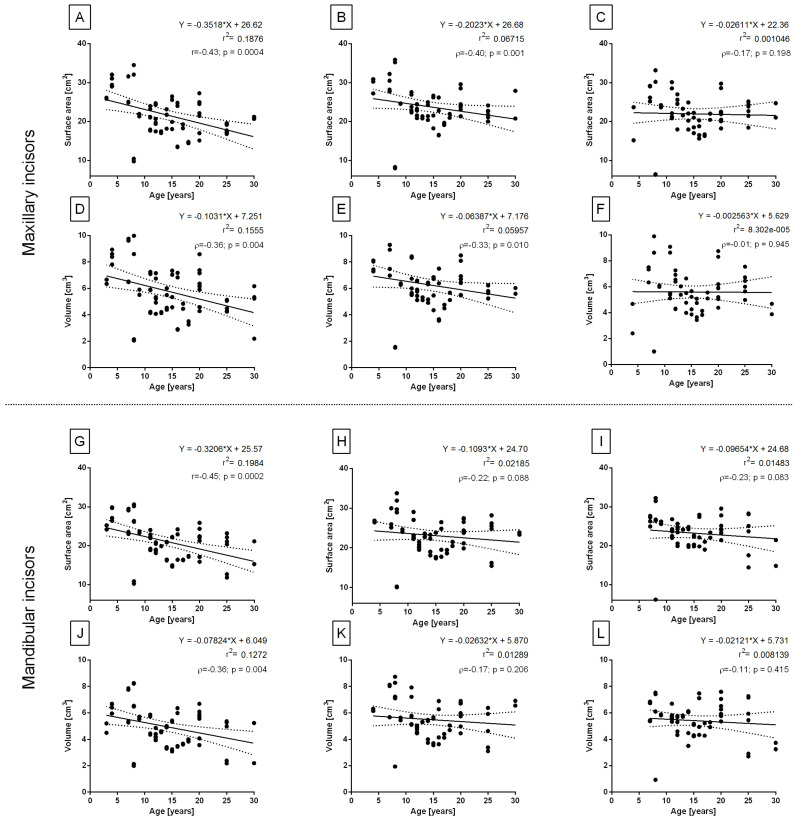
The age-related changes shown for the center (**A**,**D**,**G**,**J**), intermediate (**B**,**E**,**H**,**K**), and corner (**C**,**F**,**I**,**L**) incisors. Linear regression, Pearson’s correlation (r), and Spearman’s correlation (ρ) for the surface area (**A**–**C**,**G**–**I**) and volume (**D**–**F**,**J**–**L**) of the maxillary incisors (**A**–**F**) and mandibular incisors (**G**–**L**). The regression equations and coefficients of determination ($r^{2}$) are displayed on the plots. Values for subsequent individuals are marked with dots. Dashed lines indicate upper and lower 95% confidence intervals for the regression line. Correlation coefficients are considered significant for *p* < 0.05.

Considering the age-related changes, $r^{2}$ was low (<0.2) for both the surface area and volume of the center maxillary (Figure 9A,D) and mandibular (Figure 9G,J) incisors. For these incisors, a moderate negative correlation was noted between age and surface area (Figure 9A,G) and a weak negative correlation was noted between age and volume (Figure 9D,J). For other incisors, $r^{2}$ was low (<0.06), and a weak negative correlation was noted between age and volumetric measurements only for maxillary intermediate incisors (Figure 9B,E).

### 3.4. The EOTRH-Degree Differences in the Incisors’ Volumetric Features

**Figure 10:**
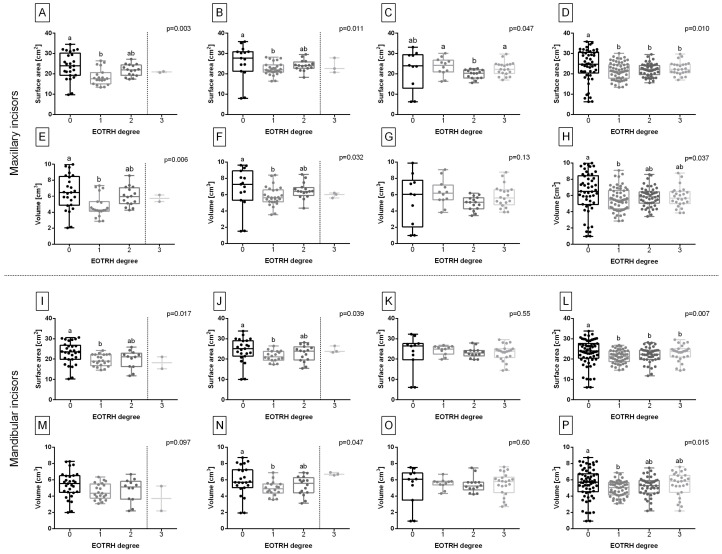
The EOTRH degree-related features compared for the center (**A**,**E**,**I**,**M**), intermediate (**B**,**F**,**J**,**N**), corner (**C**,**G**,**K**,**O**) incisors, and all incisors (**D**,**H**,**L**,**P**). The surface area (**A**–**D**,**I**–**L**) and volume (**E**–**H**,**M**–**P**) of maxillary incisors (**A**–**H**) and mandibular incisors (**I**–**P**). Data in box plots are represented by the lower quartile, median, and upper quartile, whereas whiskers represent minimum and maximum values. Values for subsequent individuals are marked with dots. The dashed line separates data sets that were not compared due to too small a number of classified incisors. Lowercase letters indicate differences between groups for *p* < 0.05.

The surface area and volume of the center and intermediate maxillary (Figure 10A,B,E,F) and mandibular incisors (Figure 10I,J,N), except the volume of the center mandibular incisors (Figure 10M), were higher for EOTRH degree 0 than degree 1. However, no differences were found between EOTRH degrees 0 and 2 as well as EOTRH degrees 1 and 2. Moreover, EOTRH degree 3 was not compared due to the too small number of classified incisors. The surface area but not the volume of the corner maxillary but not mandibular incisors was lower for EOTRH degree 2 than EOTRH degrees 1 and 3; however, no differences were found between EOTRH degrees 0 and 1 as well as EOTRH degrees 0 and 3 (Figure 10C,G,K,O).

When all incisors from different tooth positions were pooled, the surface area of maxillary and mandibular incisors was higher for EOTRH degree 0 than EOTRH degrees 1, 2, and 3 (Figure 10D,L), whereas in the same pooled group, the volume of maxillary and mandibular incisors was higher for EOTRH degree 0 than EOTRH degree 1 with no differences between EOTRH degrees 0, 2, and 3 as well as EOTRH degrees 1, 2, and 3 (Figure 10H,P). Therefore, the EOTRH degree-related differences, especially for temporary maxillary incisors, were suspected to be difficult for the clustering.

**Figure 11:**
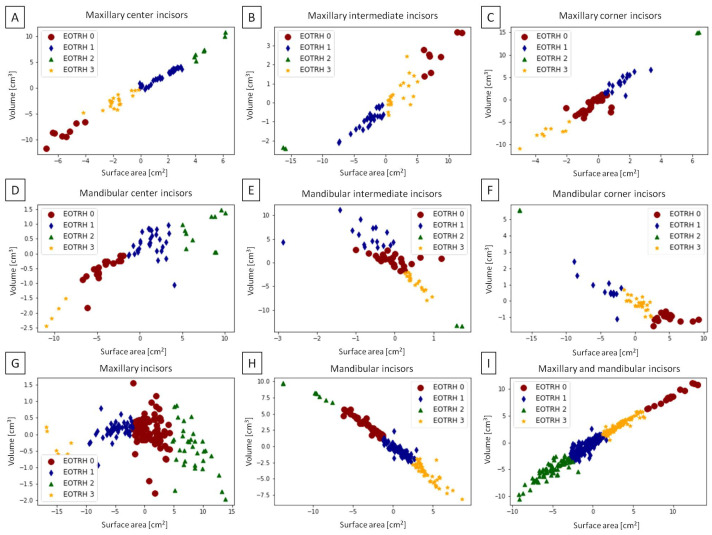
Four EOTRH degree-related classes (*EOTRH 0*, *EOTRH 1*, *EOTRH 2*, and *EOTRH 3*) extracted from the volumetric feature space for the center (**A**,**D**), intermediate (**B**,**E**), and corner (**C**,**F**) incisors in the maxilla (**A**–**C**) and mandible (**D**–**F**) as well as for all maxillary incisors (**G**), all mandibular incisors (**H**), and all maxillary and mandibular incisors (**I**). Individuals are marked with dots, rhombuses, triangles, and asterisks.

The permanent incisors’ data set was clustered as shown in Figure 11 for maxillary center incisors (Figure 11A), maxillary intermediate incisors (Figure 11B), maxillary corner incisors (Figure 11C), mandibular center incisors (Figure 11D), mandibular intermediate incisors (Figure 11E), mandibular corner incisors (Figure 11F), all maxillary incisors (Figure 11G), all mandibular incisors (Figure 11H), and all maxillary and mandibular incisors (Figure 11I). The accuracy of incisors’ classification was higher for mandibular than maxillary incisors, with the highest accuracy for mandibular center incisors. As accuracy values were >0.72 for mandibular incisors and >0.33 for maxillary incisors (Table 4), the difficult EOTRH degree-related clustering of the CT-based 3D images of equine incisors was confirmed for maxillary incisors only.

## 4. Discussion

The results of the analysis presented in this paper enabled us to demonstrate the usefulness of segmentation of the CT-based 3D equine incisors data sets. In the current study, not only the teeth volume, as in Herren et al.’s (2022) study [7], but also the surface area were used as the defining features. The volumetric features, instead of previously used angulations [5,6], were chosen for the first segmentation-based morphometric studies of equine incisors due to the close correlations shown between equine tooth volume measured by segmentation and volume measured by both water displacement and structured light scanning [7]. One may observe that the 3D Slicer used in this study was successfully used for manual segmentation of a mandible [36] and femoral head [3]. In both studies, the 3D Slicer outperformed other available segmentation software. Thus, the image processing approach proposed in this study may be considered actual and promising.

In the first step of this paper, the axial vertical symmetry of the equine incisors was evidenced by the lack of side-related differences, high values of the coefficients of determination, and very strong positive side-relation correlations. These findings allow not only to pool the data of the right and left sides, which significantly improves the readability of the results, but can also be used in further clinical applications. For example, the volumetric measures of equine incisors may be compared before and after teeth correction to assess occlusion improvement [5,6,25]. Moreover, the effect of unilateral incisor malocclusions and/or diseases [17,20,23] on occlusion, mastication, and attrition may be assessed. Our observations are consistent with the previous ones concerning equine cheek teeth, for which no differences between the right and left sides of the maxilla and mandible were shown [7]. Contrary to the axial vertical symmetry, no evidence of the axial horizontal symmetry of the equine incisors was shown. The surface area and volume of the center incisors were generally higher in the maxilla than mandible, similar to previously reported cheek teeth [7]. Interestingly, the intermediate and corner temporary incisors were slightly larger in the mandible than in the maxilla, which is difficult to compare with previous studies because the available literature lacks volumetric measurements of the equine temporary incisors.

### 4.1. The Eruption-Related Differences in the Incisors’ Volumetric Features

Against the deficiencies in the available literature, this study demonstrated in a quantifiable way that permanent incisors showed higher volumetric measurements than temporary ones. These results were expected, as the equine permanent teeth are larger and longer than temporary ones [26]. However, the evidence of easy eruption-related clustering of the CT-based 3D images was suspected but not so obvious anymore. Confirmation of the easy classification of teeth that are significantly different in size may be useful in an assessment of the incisors’ malocclusions and/or diseases, especially concerning oligodontia and fractures with reduced tooth volume [17,20,23].

### 4.2. The Location-Related and Age-Related Differences in the Incisors’ Volumetric Features

In this study, the location-related differences were more easily accessible for temporary incisors than permanent ones. However, some differences in the surface area and volume in older horses were perceptible. One may observe that the incisors of mature horses display a convexly curved surface [27,37]. This curvature is more easily visible in the center and intermediate than the corner incisors [5]; however, no differences between maxillary and mandibular incisors were reported. As the subject curvature changes with advancing age [37], both location- and age-related volumetry measures should be considered jointly, especially because of incisors’ angular changes and also their change in length with advancing age [27].

One may note that the extracted volumetric features were sufficient for moderate classification of teeth that were significantly different in location; however, a similar protocol concerning age groups could not be carried out. Due to the small number of individuals in the consecutive age groups [20], age-related changes were shown using linear regression and correlation coefficients following Kau et al. (2020) [5] and clustering was not performed. The problem of the small number of examined teeth is discussed in the Limitations Section.

### 4.3. The EOTRH Degree-Related Differences in the Incisors’ Volumetric Features

In recent volumetric studies, the volumes of equine cheek teeth without tooth pathology were measured [7], whereas in this volumetric study and the previous cephalometric one [5], the EOTRH syndrome was also considered. Since EOTRH is an age-related disease [19], for research in which the inclusion criterion was age of at least 10 years [32], the EOTRH degree-related differences were shown for permanent teeth only.

Radiological signs and EOTRH degrees tend to differ by incisor location [38]. It has been shown that corner incisors most often show clinical and radiographic signs of EOTRH [39]. Corner incisors are suspected to receive higher biomechanics due to the lack of bilateral support from neighboring incisors [32,40]. However, in this study, the surface area and volume of the corner incisor did not differ from the other two considering EOTRH degrees. Therefore, one may note that the extracted volumetric features were not sufficient for full differentiation of the EOTRH degrees. Despite the classification of EOTRH degrees being quite good and considered moderately difficult for the mandibular incisors, the classification metrics for the maxillary incisors were poor. Therefore, in addition to simple volumetric features, more informative features should be included in the K-means clustering. As the K-means clustering successfully operates on multidimensional spaces [41], not just a two-dimensional set as in this research, the inclusion of the additional feature may improve the classification metrics and thus the efficiency of disease radiological signs recognition. Among the potentially useful features in future research, incisor angulation [5,6] and image texture features [29,33] can be included in the multidimensional spaces of EOTRH-degree determination.

### 4.4. Limitations

The manual image segmentation could result in variations in teeth volume over- or underestimation, which depended on the skill and experience of the observer [7]. In the current study, the single-observer protocol was used for the CT image segmentation; thus, the observer-related error is unknown. In further studies, the double-blinded-observer protocol as well as semi-automatic and automatic segmentation protocols should be evaluated. Moreover, in further studies, the performance metrics of a medical image segmentation should be used to indicate the perceptual quality of segmentation, especially when the AI-based method for image segmentation is used. Performance metrics, among which the most commonly used are the Dice score and Jaccard index, are implemented for error risk minimization [42]. In this study, since manual segmentation rather than an AI-based method is used, performance metrics may be considered as advisable and informative but not obligatory. One may observe that the current research is not the only study on CT image morphometry/segmentation where a single observer performs the annotations without performance metrics’ reporting. Similar protocols were used by Listmann et al. (2017) [6], Kau et al. (2020) [5], Herren et al. (2022) [7], and Mandolini et al. (2022) [3]. Nevertheless, the current study should be considered preliminary.

Another limitation is the relatively small sample size. In this study, 582 incisors in 49 horse heads were segmented and measured. Although this is more than 216 incisors in 18 heads [6] and 120 cheek teeth in 5 heads [13], and comparable to 576 incisors in 48 heads [5], it is still not enough to properly assess age-dependent differences. A larger number of equine incisors should be segmented and volumetrically measured to consider the impact of age, horse size, gender, and breed on the returned features. Even more importantly, the dental history, including teeth correction and treatment, should be considered. Obtaining such data at the slaughterhouse is impossible or very difficult; therefore, apart from postslaughter inspections, efforts should be made to implement volumetric measurements on clinical data, as were performed by Kau et al. (2020) [5]. Only a big-data clinical study will provide complete data on the marked dynamic changes in the equine dentition [7] and occlusion [6] throughout a horse’s life.

### 4.5. Further Directions

In further studies, a comparison of the segmentation results from various segmentation approaches, following Herren et al. (2022) [7] and Mandolini et al. (2022) [3], is needed. As semi-automatic and automatic algorithms rely on greyscale values of adjacent pixels, automatic segmentation seems to be challenging [7]. Therefore, the delineation of a distinct contour by using image filtering may be a good direction in the development of radiological image segmentation. In human dentistry, radiographs are commonly filtered [43,44]. In equine dentistry, the first radiograph filtering protocols have also been applied [29,33]. As filtering enhances brightness, contrast, and edges, they are applied to remove outliers, increase contrast, preserve edges, highlight regions of rapid intensity change, and remove noise [33,43,44]. Since radiograph filtering has already been used together with textural analysis, both in humans [44] and horses [33], texture features’ extraction may be a promising direction for further CT image processing.

The intention to guide future research is also to introduce 3D segmentation of CT data sets and volumetric measurements to equine clinical practice. However, the opinions of researchers differ. Herren et al. (2022) think that the semi-automatic measurement of tooth volume will most likely not reach a widespread clinical application [7], while Kau et al. (2020) believe that 3D tooth morphometry will become clinically applicable [5]. Moreover, the use of 3D segmentation has been suggested to remain limited to research facilities due to the required resources and skills [45,46]. However, after performing this preliminary study, this remark can be rejected. Considering the large number of CT examinations of horses’ heads that we perform in the Equine Clinic at WULS, the high quality of the multidetector CT images, and the rapid technological progress of CT image processing, we agree with the opinion of Kau et al. (2020) and also believe in clinical progress, especially since the multidetector CT imaging of a standing, sedated horse is the main direction in the development of advanced imaging diagnostics of the equine head [16,28].

## 5. Conclusions

The CT-based 3D images of equine incisors can be successfully segmented using the routinely achieved multidetector CT data sets and the proposed data processing approaches. The returned volumetric features differed between eruption-related as well as partially location-related and EOTRH degree-related groups, thus enabling easy clustering of erupted incisors, moderate clustering tooth location, and EOTRH degree in the mandible, as well as difficult clustering of EOTRH degree in the maxilla.

## Figures and Tables

**Table 1:** The number of incisors (*n*) and the age of the horses (mean ± SD years) in the subsequent studied groups.

Incisors	Maxilla	Mandible
Temporary		
Center	34 (1.88±0.33)	34 (1.88±0.33)
Intermediate	36 (1.94±0.42)	38 (2.05±0.62)
Corner	38 (2.05±0.62)	40 (2.15±0.75)
Permanent		
Center	62 (14.26±6.71)	64 (14.06±6.70)
Intermediate	60 (14.63±6.49)	60 (14.77±6.30)
Corner	58 (15.00±6.28)	58 (15.14±6.07)
Total	288	294

**Table 2:** The classification metrics (number of samples (n), Recall, Precision, F1-score, Accuracy) of K-means clustering into two specific eruption-related classes (*Temporary* and *Permanent*). Metrics calculated for the center, intermediate, and corner incisors of the maxilla and mandible (in total n = 582 incisors).

Incisors	Maxilla					Mandible				
Metrics	n	Recall	Precision	F1	Accuracy	n	Recall	Precision	F1	Accuracy
Center										
Temporary	34	1.00	0.60	0.75	0.76	34	1.00	0.72	0.84	0.87
Permanent	62	0.63	1.00	0.77		64	0.80	1.00	0.89	
Intermediate										
Temporary	36	1.00	0.64	0.78	0.79	38	1.00	0.83	0.90	0.92
Permanent	60	0.67	1.00	0.80		60	0.87	1.00	0.93	
Corner										
Temporary	38	1.00	0.68	0.81	0.81	40	1.00	0.74	0.85	0.86
Permanent	58	0.69	1.00	0.82		58	0.76	1.00	0.86	

**Table 3:** The classification metrics (number of samples (n), Recall, Precision, F1-score, Accuracy) of K-means clustering into three specific location-related classes (*Center*, *Intermediate*, and *Corner*). Metrics calculated for the temporary and permanent incisors of the maxilla and mandible (in total n = 582 incisors).

Incisors	Maxilla					Mandible				
Metrics	n	Recall	Precision	F1	Accuracy	n	Recall	Precision	F1	Accuracy
Temporary										
Center	34	0.71	1.00	0.83	0.86	34	1.00	0.92	0.96	0.93
Intermediate	36	1.00	0.71	0.83		38	0.92	0.88	0.90	
Corner	38	0.87	1.00	0.93		40	0.88	1.00	0.93	
Permanent										
Center	62	1.00	0.61	0.76	0.69	64	0.73	1.00	0.85	0.76
Intermediate	60	0.33	0.56	0.42		60	1.00	0.58	0.74	
Corner	58	1.00	0.72	0.84		58	0.55	1.00	0.71	

**Table 4:** The classification metrics (number of samples (n), Recall, Precision, F1-score, Accuracy) of K-means clustering into four specific EOTRH degree-related classes (*EOTRH 0*, *EOTRH 1*, *EOTRH 2*, and *EOTRH 3*). Metrics calculated for the center, intermediate, and corner incisors of the maxilla and mandible as well as all maxillary incisors, all mandibular incisors, and all maxillary and mandibular incisors (total) (in total n = 362 permanent incisors).

Incisors	Maxilla					Mandible				
Metrics	n	Recall	Precision	F1	Accuracy	n	Recall	Precision	F1	Accuracy
Center										
*EOTRH 0*	25	0.32	1.00	0.48	0.34	29	0.66	1.00	0.79	0.80
*EOTRH 1*	19	0.58	0.39	0.47		21	0.95	0.67	0.78	
*EOTRH 2*	16	0.00	0.00	0.00		12	0.83	0.91	0.87	
*EOTRH 3*	2	1.00	0.11	0.19		2	1.00	0.50	0.67	
Intermediate										
*EOTRH 0*	14	0.57	1.00	0.73	0.57	23	1.00	0.96	0.98	0.72
*EOTRH 1*	27	0.85	0.79	0.82		18	0.89	1.00	0.94	
*EOTRH 2*	16	0.00	0.00	0.00		16	0.06	0.50	0.11	
*EOTRH 3*	3	1.00	0.14	0.25		3	1.00	0.17	0.29	
Corner										
*EOTRH 0*	10	1.00	0.36	0.53	0.33	11	1.00	0.73	0.85	0.72
*EOTRH 1*	12	0.00	0.00	0.00		11	0.64	0.54	0.58	
*EOTRH 2*	16	0.00	0.00	0.00		14	0.14	1.00	0.25	
*EOTRH 3*	20	0.45	1.00	0.62		22	1.00	0.79	0.88	
All										
*EOTRH 0*	49	1.00	0.54	0.70	0.47	63	0.78	1.00	0.88	0.74
*EOTRH 1*	58	0.28	0.32	0.30		50	1.00	0.61	0.76	
*EOTRH 2*	48	0.29	0.42	0.35		42	0.19	1.00	0.32	
*EOTRH 3*	25	0.24	1.00	0.39		27	1.00	0.63	0.77	
Total										
*EOTRH 0*	112	0.14	1.00	0.25	0.54					
*EOTRH 1*	108	0.77	0.46	0.58						
*EOTRH 2*	90	0.49	0.64	0.55						
*EOTRH 3*	52	1.00	0.53	0.69						

## Data Availability

In order to facilitate the replication of the experiments presented in this work, the data set (data set location: https://zenodo.org/records/8327594 (accessed on 7 September 2023)) and the experimental source code are made available to the public under an open license.
